# Epigenetic and Coping Mechanisms of Stress in Affective Disorders: A Scoping Review

**DOI:** 10.3390/medicina60050709

**Published:** 2024-04-25

**Authors:** Brenda-Cristiana Bernad, Mirela-Cleopatra Tomescu, Teodora Anghel, Diana Lungeanu, Virgil Enătescu, Elena Silvia Bernad, Vlad Nicoraș, Diana-Aurora Arnautu, Lavinia Hogea

**Affiliations:** 1 Doctoral School, “Victor Babes” University of Medicine and Pharmacy from Timișoara, 300041 Timișoara, Romania; bernad.brenda@umft.ro; 2 Center for Neuropsychology and Behavioral Medicine, “Victor Babes” University of Medicine and Pharmacy from Timișoara, 300041 Timișoara, Romania; anghel.teodora@umft.ro (T.A.); hogea.lavinia@umft.ro (L.H.); 3 Multidisciplinary Heart Research Center, “Victor Babes” University of Medicine and Pharmacy, 300041 Timișoara, Romania; tomescu.mirela@umft.ro (M.-C.T.); aurora.bordejevic@umft.ro (D.-A.A.); 4 Department of Internal Medicine, ”Victor Babes” University of Medicine and Pharmacy, 300041 Timișoara, Romania; 5 Timisoara Municipal Clinical Emergency Hospital, 300040 Timișoara, Romania; 6 Department of Neuroscience, “Victor Babes” University of Medicine and Pharmacy from Timișoara, 300041 Timișoara, Romania; enatescu.virgil@umft.ro; 7 Center for Modeling Biological Systems and Data Analysis, “Victor Babes” University of Medicine and Pharmacy from Timișoara, 300041 Timișoara, Romania; dlungeanu@umft.ro; 8 Department of Functional Sciences, Faculty of Medicine, “Victor Babes” University of Medicine and Pharmacy from Timișoara, 300041 Timișoara, Romania; 9 Clinic of Psychiatry, “Pius Brinzeu” County Clinical Emergency Hospital, 300723 Timișoara, Romania; 10 Department of Obstetrics and Gynecology, “Victor Babes” University of Medicine and Pharmacy from Timișoara, 300041 Timișoara, Romania; 11 Ist Clinic of Obstetrics and Gynecology, “Pius Brinzeu” County Clinical Emergency Hospital, 300723 Timișoara, Romania; vnicoras@gmail.com; 12 Center for Laparoscopy, Laparoscopic Surgery and In Vitro Fertilization, “Victor Babes” University of Medicine and Pharmacy, 300041 Timișoara, Romania; 13 Institute of Cardiovascular Diseases Timișoara, 300310 Timișoara, Romania

**Keywords:** epigenetic, coping, stress, affective disorders, DNA methylation, childhood trauma, stressor, depressive disorder, dietary deficiencies

## Abstract

This review aims to explore the intricate relationship among epigenetic mechanisms, stress, and affective disorders, focusing on how early life experiences and coping mechanisms contribute to susceptibility to mood disorders. Epigenetic factors play a crucial role in regulating gene expression without altering the DNA (deoxyribonucleic acid) sequence, and recent research has revealed associations between epigenetic changes and maladaptive responses to stress or psychiatric disorders. A scoping review of 33 studies employing the PRISMA-S (Preferred Reporting Items for Systematic Reviews and Meta-Analyses—Statement) guidelines investigates the role of stress-induced epigenetic mechanisms and coping strategies in affective disorder occurrence, development, and progression. The analysis encompasses various stress factors, including childhood trauma, work-related stress, and dietary deficiencies, alongside epigenetic changes, such as DNA methylation and altered gene expression. Findings indicate that specific stress-related genes frequently exhibit epigenetic changes associated with affective disorders. Moreover, the review examines coping mechanisms in patients with bipolar disorder and major depressive disorder, revealing mixed associations between coping strategies and symptom severity. While active coping is correlated with better outcomes, emotion-focused coping may exacerbate depressive or manic episodes. Overall, this review underscores the complex interplay among genetic predisposition, environmental stressors, coping mechanisms, and affective disorders. Understanding these interactions is essential for developing targeted interventions and personalized treatment strategies for individuals with mood disorders. However, further research is needed to elucidate specific genomic loci involved in affective disorders and the clinical implications of coping strategies in therapeutic settings.

## 1. Introduction

### 1.1. Epigenetic Mechanisms—General Overview

Epigenetic mechanisms orchestrate a complex symphony of gene regulation, influencing cellular functions, development, and responses to environmental stimuli. At the core of epigenetics lies dynamic modifications to DNA and histone proteins, along with the intricate interplay of non-coding RNAs, collectively shaping gene expression patterns without altering the underlying DNA sequence. Epigenetic factors refer to functional changes in the genome without changes in the DNA sequence. Such modifications regulate gene expression and phenotypes, for example, through mechanisms, such as DNA methylation. Epigenetic differences may be a consequence of exposure to stress-related factors during critical periods of development and, therefore, contribute to susceptibility to certain psychiatric disorders. Recent studies have shown associations between specific epigenetic changes and the risk of maladaptive responses to stress or mental disorders [1].

Genes do not function as fixed patterns; their expression is regulated dynamically and often reversibly. Epigenetic molecular elements are cell chromatin, dynamic DNA, histone, and microRNA changes [2].

DNA Methylation: One of the most extensively studied epigenetic modifications, DNA methylation involves the addition of methyl groups to cytosine bases within CpG dinucleotides. This modification typically represses gene transcription by impeding the binding of transcription factors or recruiting methyl-binding proteins that induce chromatin condensation. Conversely, DNA demethylation processes, mediated by enzymes, such as TET proteins, facilitate gene activation and transcriptional plasticity.

Histone Modifications: Histone proteins, around which DNA is wrapped to form chromatin, undergo various post-translational modifications, including acetylation, methylation, phosphorylation, and ubiquitination. These modifications alter the chromatin structure, thereby modulating gene accessibility and transcriptional activity. For instance, histone acetylation generally correlates with transcriptional activation, while histone methylation can either activate or repress gene expression depending on the specific histone residues and the degree of methylation.

Non-coding RNAs: Non-coding RNAs, including microRNAs (miRNAs) and long non-coding RNAs (lncRNAs), play crucial roles in epigenetic regulation by modulating gene expression at post-transcriptional and transcriptional levels, respectively. miRNAs can bind to complementary sequences within target mRNAs, leading to mRNA degradation or translational repression, thereby fine-tuning gene expression. Similarly, lncRNAs can interact with chromatin-modifying complexes to regulate gene transcription, chromatin organization, and epigenetic inheritance [2].

Affective disorders tend to be both recurrent and progressive, in the sense that the episodes follow one another after shorter remission intervals or with an increased rate of cyclicity [3,4]. About 70% of patients who had their first episode of unipolar depression will later have multiple episodes, and almost all bipolar disorders are recurrent [5].

Numerous studies have shown that psychosocial factors can precipitate depression, as well as mania [6,7]. However, most people who are exposed to stressful events do not develop a psychiatric disorder [8,9,10,11]. This begs the question, why do some people develop an emotional disorder about a stressful life event and others do not? The answer to this question is complex; it involves genetic predisposition, personality, previous experiences, family distress, adequate social support network, and probably the individual’s response to stress [12].

### 1.2. Epigenetic Insights into Depression

Depression, a pervasive psychiatric disorder characterized by persistent sadness, lethargy, and impaired cognition, is intricately linked to dysregulated epigenetic processes. Emerging evidence suggests that epigenetic modifications contribute to altered neuroplasticity, disrupted neurotransmitter signaling, and aberrant stress responses observed in individuals with depression.

DNA Methylation Dynamics: Dysregulated DNA methylation patterns have been implicated in depression pathogenesis, particularly within genes involved in hypothalamic–pituitary–adrenal (HPA) axis regulation, neurotrophic signaling, and synaptic plasticity. For instance, hypermethylation of the glucocorticoid receptor (GR) gene promoter has been associated with reduced GR expression and HPA axis hyperactivity, contributing to dysregulated stress responses in depression [13].

Histone Modifications and Neuroplasticity: Altered histone acetylation and methylation profiles have been reported in depression, influencing chromatin accessibility and the transcriptional regulation of genes critical for neuroplasticity and mood regulation. For example, reduced histone acetylation at the promoters of neurotrophic factor genes, such as brain-derived neurotrophic factor (BDNF), may compromise synaptic plasticity and neuronal survival pathways implicated in depression pathophysiology [14].

Non-coding RNAs as Epigenetic Regulators: The dysregulated expression of miRNAs and lncRNAs has been observed in depression, impacting the expression of target genes involved in neurotransmitter synthesis, synaptic transmission, and neuroinflammatory processes. The perturbed miRNA-mediated regulation of serotonin transporter (SERT) expression, for instance, may contribute to the altered serotonin signaling implicated in depression pathogenesis [3].

### 1.3. Epigenetic Insights into Bipolar Disorder

Bipolar disorder, characterized by recurrent episodes of mania and depression, encompasses the multifaceted interplay of genetic predisposition and environmental influences, modulated in part by epigenetic mechanisms. Understanding the epigenetic dysregulation underlying bipolar disorder offers valuable insights into its etiology and potential avenues for therapeutic intervention.

Dynamic DNA Methylation Patterns: Epigenome-wide association studies (EWASs) have revealed aberrant DNA methylation patterns associated with bipolar disorder susceptibility and mood state transitions. The differential methylation of genes involved in neurotransmitter signaling, circadian rhythm regulation, and synaptic plasticity may contribute to the pathophysiology of bipolar disorder [15].

Histone Modifications and Mood Regulation: Dysregulated histone modifications, including the altered acetylation and methylation profiles, have been implicated in bipolar disorder pathogenesis, influencing the expression of genes critical for mood regulation, circadian rhythm synchronization, and synaptic plasticity. The modulation of histone deacetylase (HDAC) activity, for instance, may represent a potential therapeutic strategy for restoring mood stability in bipolar disorder [15].

Non-coding RNAs in Bipolar Disorder Pathophysiology: The perturbed expression of miRNAs and lncRNAs has been implicated in bipolar disorder, impacting the expression of genes involved in neurotransmitter metabolism, ion channel function, and synaptic plasticity. The dysregulated miRNA-mediated regulation of genes associated with glutamatergic and GABAergic neurotransmission may contribute to mood instability and cognitive dysfunction in bipolar disorder [15].

### 1.4. Coping Mechanisms of Stress in Affective Disorders

In the context of affective disorders, coping mechanisms play a crucial role in modulating the impact of stressors on psychological well-being and mental health outcomes. Coping refers to the cognitive and behavioral efforts individuals employ to manage stress, adversity, or challenging situations. Understanding how coping mechanisms interact with epigenetic processes provides valuable insights into the etiology, recurrence, and progression of mood disorders, such as depression and bipolar disorder.

Research has shown that coping mechanisms can influence the expression of genes involved in stress response pathways and contribute to individual differences in vulnerability or resilience to affective disorders. Preclinical animal studies have shown that exposure to stress is associated with changes in the epigenome (e.g., changes in the genes involved in the stress response), as well as the occurrence of depression-like behavior; similarly, chronic exposure to unpredictable stress alters histone acetylation in the forebrain, with the appearance of anxiety-like behavior and increased vulnerability to stress, as well as cognitive deficits. In the case of animals that were subjected to stress from the first days of life and separation from their mother, they showed depressive behaviors in adulthood [16].

Moreover, studies conducted on the families of patients with major depressive disorder or bipolar disorder have shown that there is a greater segregation of these affective disorders among family members [17,18]. The transmission rate to first-degree relatives for type I bipolar disorder is between 4 and 24%, for type II bipolar disorder is between 1 and 5%, and for major depressive disorder is between 4 and 24% [19]. Identical values were also described in schizophrenia and schizoaffective disorder. The transmission rates to relatives highlight the complex interplay between genetic and environmental factors in shaping susceptibility to mood disorders.

However, empirical data have highlighted the significant interindividual differences in the response to stress and adversity. Some authors speak of the “invulnerable child”, referring to the positive interaction of events that determine the child’s resilience (invulnerability) to adversity [20]. A simple interpretation of such a phenomenon is the interaction between genetic and environmental factors that ultimately determines susceptibility. The interaction between coping mechanisms and epigenetic processes offers a nuanced understanding of how stressors influence gene expression patterns, neurobiological pathways, and ultimately, mental health outcomes. Investigating the psychological mechanisms underlying coping strategies and their epigenetic correlates can inform personalized interventions aimed at enhancing resilience, mitigating stress-related risk factors, and promoting mental well-being in individuals vulnerable to affective disorders. The need to translate the possible genetic influences of individual vulnerability into psychological mechanisms remains. This review aims to analyze clinical trials on coping mechanisms and their interaction with epigenetic mechanisms in patients with mood disorders. The effect of the interaction between the two mechanisms on these disorders’ onset, recurrence, and progression was mainly followed in the review.

## 2. Materials and Methods

**Figure 1:**
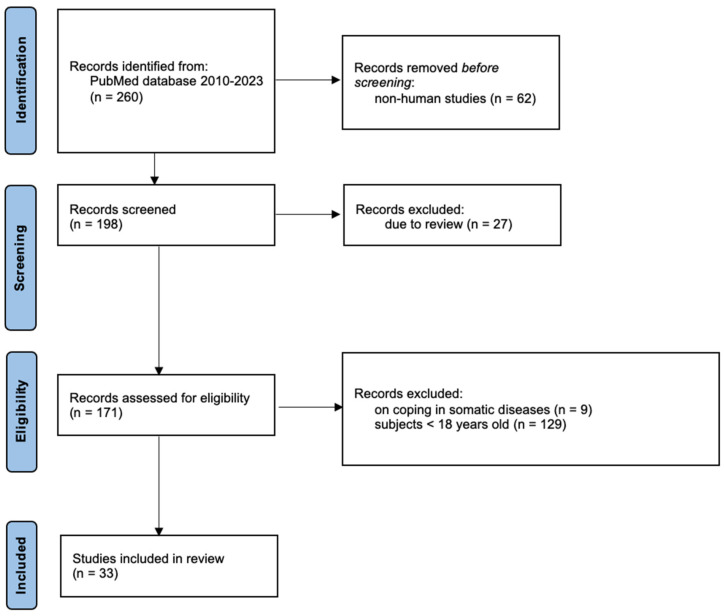
Selection process in the PRISMA flow diagram.

According to PRISMA-S guidelines, 33 studies were reviewed in this article (Figure 1). These studies investigated both the role of epigenetic mechanisms induced by stressors in the occurrence/development and/or progression of affective disorders and the role of stress-adaptation mechanisms in these disorders. Stress factors include childhood trauma, stress at work, dietary deficiencies, exposure to chemicals, and so on. Inclusion has not been limited to studies examining specific types of stressors.

Regarding epigenetic changes, they were defined to include any valid indicator of epigenetic changes (e.g., DNA methylation, DNA acetylation, altered transcription, direct changes in gene expression).

Inclusion criteria were as follows:

- studies in which a valid measurements of epigenetic changes that were associated with a significant stress factor (death of a loved one, emotional, physical, sexual abuse, etc.) were performed;
- studies that assessed whether the epigenetic change was correlated with a diagnosis of an affective disorder;

The exclusion criteria were as follows:

- subjects with somatic comorbidities (any physical health conditions or diseases that coexist alongside the psychiatric disorders);
- subjects under the age of 18 years old.

The studies were identified by searching the PubMed database between 2010 and 2023, using the following keywords: epigenetic mechanisms, stressors, major depressive disorder, suicidal ideation, bipolar disorder, mania, and coping results.

## 3. Results

Thirty-three articles were screened that followed the relationship between an epigenetic change associated with stress and the diagnosis of affective disorder, as well as the relationship among a stress factor, induced epigenetic changes, and the evaluation of psychiatric symptoms (suicidal ideation, manic states, etc.). DNA methylation, in particular, was the change evaluated in these studies. Moreover, despite the broad meaning of the stressors, the identified studies assessed the adversity of early childhood experiences (abuse, abuse, early loss of a parent).

Epigenetic changes in the following stress-associated genes have been frequently linked to the diagnosis of affective disorder (Table 1):

- NR3C1 (human glucocorticoid receptor gene);
- SLC6A4 (serotonergic transporter gene);
- BDNF (brain-derived neurotrophic factor);
- FKBP5 (FK506 5 binding protein gene);
- SKA2 (kinetochore protein gene);
- OXTR (oxytocin receptor) and genes encoding oligodendrocytes.

*NR3C1 (human glucocorticoid receptor gene).* Bustamante and co-workers [21] reported significantly higher scores on the scale of childhood abuse and trauma screening in the group of patients diagnosed with recurrent depressive disorder (*n* = 76) compared to the healthy control group (*n* = 76). Furthermore, methylation of the NR3C1 gene could be predicted by a history of childhood abuse or major depressive disorder. A history of childhood abuse has been associated with increased methylation at the NR3C1 transcription-factor-binding site, leading to reduced NR3C1 gene expression; major depressive disorder has been associated with low methylation at the downstream locus, indicating that NR3C1 gene expression is unchanged.

Radtke et al. [22] analyzed the association between NR3C1 methylation (at 41 CpG sites) and depressive symptoms in individuals with a history of childhood abuse (*n* = 46). No significant association was found between NR3C1 methylation and depressive symptoms. However, correlations have been found between the methylation of two CpG loci located in the NR3C1 gene promoter and the specific symptoms of depression.

Perroud et al. [23] described positive correlations between NR3C1 methylation and childhood abuse in patients with major depressive disorder (*n* = 99). The level of NR3C1 methylation was correlated with the form of abuse (physical abuse, sexual abuse, emotional neglect) and its severity.

De Assis Pinheiro et al. [24] showed that alcohol consumption, overweight, and high cortisol levels are related to NR3C1 non-methylation, while depression is related to its methylation (*n* = 386).

*FKPB2 (FK506 5 binding protein gene).* Weder et al. [26] compared children with a history of abuse (*n* = 94) and no history of abuse (*n* = 96), noting significant differences in NR3C1 methylation at six CpG sites in the promoter region. The researchers reported that methylation at two of these CpG sites was able to predict the onset of psychiatric symptoms. The study also looked at FKBP5 gene methylation in these groups and found positive correlations between gene methylation in abused children vs. the abused. Significant differences were found between the two groups regarding BDNF methylation.

On the other hand, Tyrka et al. [27] reported significantly lower NR3C1 methylation across the promoter region and at six other CpG sites in people with a history of childhood abuse and/or major depressive disorder, bipolar disorder, or generalized anxiety disorder. This result contradicts previous studies, suggesting a much greater complexity in regulating the NR3C1 gene.

Also, Flasbeck and Brüne [28] demonstrated that FKBP5 was associated with anxiety and reduced empathy. Despite expectations, there was no discernible impact of childhood maltreatment on DNA methylation. Additionally, no methylation distinctions were evident between a clinical group and a non-clinical group concerning FKBP5. However, there was a slight discrepancy in NR3C1 methylation levels, although its biological significance is questionable.

*SLC6A4 (serotonergic transporter gene).* Sanwald et al. [29] concluded that SLC6A4 methylation was not related to depression severity, age at depression onset, or SLEs in the entire group but positively related to depression severity in women (*n* = 95).

Swartz and colleagues [30] operationalized environmental stress, using the adolescent socioeconomic status as a measurement method. The researchers found that a poor socioeconomic status was associated with higher methylation of SLC6A4, which may lead to the worsening of depressive symptoms. These findings suggest that adolescent stress may contribute to the severity of the disease through epigenetic changes in the SLC6A4 gene.

Booij et al. [31] compared individuals with a diagnosis of major depressive disorder (*n* = 33) and the healthy control group (*n* = 36), reporting insignificant differences in SLC6A4 methylation. However, the authors found positive correlations between the history of childhood abuse and SLC6A4 methylation.

In another study that operationalized environmental stress, Lei et al. [32] used the crime rate in the neighborhood as a measure. This correlated positively with methylation of the SLC6A4 promoter, but only in individuals carrying the short allele gene. These findings suggest that the gene’s interaction with environmental factors may interact in a genotype-dependent manner.

Kang and colleagues [33] analyzed the association among childhood adversity, the severity of depressive symptoms, and SLC6A4 methylation in patients with major depressive disorder (*n* = 108). Thus, higher methylation has been reported in patients with severe symptoms and a history of childhood abuse.

On the other hand, Alasaari et al. [34] reported significantly lower SLC6A4 promoter methylation in nurses (*n* = 24) compared to other areas with a low stress level (*n* = 25). These results contradict the results of previous studies, which support a positive association between stress and SLC6A4 methylation.

In 2023, Comtois-Cabana et al. [25] investigated the association between depressive symptoms and the methylation levels of specific genes, including NR3C1 and SLC6A4. Adults with higher depressive symptoms exhibited higher methylation levels at two CpG sites across the NR3C1 promoter regions (*n* = 34) and lower methylation levels at three CpG sites across the SLC6A4 promoter region (*n* = 31). This study is the first to investigate the association between depressive symptoms and NR3C1 methylation levels in saliva samples of adults. The findings are consistent with some previous studies that also detected higher levels of NR3C1 methylation in blood samples of depressed adults compared to controls. While the majority of studies have found higher SLC6A4 methylation levels in association with depressive symptoms, some studies have reported conflicting results.

Song et al. [35] analyzed the correlations among BDNF methylation, scale scores for depressive symptoms, and work stress in the Japanese population (*n* = 774). Significantly lower BDNF methylation was reported in individuals with high questionnaire scores but was considerably higher in individuals with high work stress levels.

Studies by Sadeh et al. [36,37] investigated the relationship among post-traumatic stress, depression, and SKA2 gene methylation in a group of war veterans. In the first study, a positive association between PTSD symptoms and SKA2 methylation was reported, but there was no association with depressive symptoms (*n* = 145). On the other hand, in a subsequent study, Sadeh and colleagues reported an association between PTSD and SKA2 methylation, as well as an association between gene methylation and depressive symptoms (*n* = 466). The authors acknowledged that the discrepancy was due to chronic PTSD (i.e., the length of time since PTSD was diagnosed).

*OXTR (oxytocin receptor).* Smearman et al. [38] reported a positive association between a history of childhood abuse and OXTR methylation; however, the association was no longer valid after the correction for multiple comparisons.

Ludwig B. et al. [40] suggested a positive but nonsignificant association between the severity of depression symptoms and OXTR methylation. Also, the severity of emotional neglect in patients with affective disorders, but not childhood adverse experiences, was associated with OXTR methylation levels. On the other hand, Reiner et al. [42] found significantly lower exon 1 OXTR DNA methylation in depressed patients compared to healthy controls both before and after treatment. This suggests that lower methylation at CpG sites is associated with higher transcriptional activity of the OXTR gene, potentially leading to increased oxytocin receptor expression in the brain areas implicated in depression.

Kogan et al. [41] demonstrated that contextual stressors, both in childhood and emerging adulthood, can increase defensive/hostile relational schemas. These schemas, in turn, are linked to substance abuse and depressive symptoms. Interestingly, the study also explored the moderating role of DNA methylation in the OXTR gene. When OXTR DNA methylation levels were high, the association between contextual stress and defensive/hostile relational schemas was exacerbated. Conversely, when OXTR DNA methylation levels were low, contextual stress did not significantly influence defensive/hostile schemas.

Regarding specific coping strategies, none of the studies thoroughly answered whether they are predictive of affective disorders. However, in most studies, emotion-focused coping was associated with the recurrence of depressive or manic episodes. These strategies could also be associated with a longer recovery time. Relatively few bipolar disorder studies have been identified, with findings mainly limited to major depressive disorder. In cross-sectional studies, a clear distinction between psychiatric symptoms and emotion-focused coping strategies is difficult to achieve, so the results of these studies can only partially address the goal (Table 2).

Longitudinal studies have consistently demonstrated the pivotal role of coping strategies in determining the trajectory of affective disorders. Research by Fletcher et al. [45], Horwitz et al. [44], and Kasi et al. [43] highlight that adaptive coping strategies are associated with more extended remission periods and a reduced risk of recurrence. Conversely, passive coping is linked to an increased risk of recurrence and more severe symptoms of depression. Additionally, while individuals diagnosed with major depressive disorder may not significantly differ from their healthy counterparts in their response to stressful situations [50], problem-centered coping emerges as a good predictor of post-hospital symptoms [51].

## 4. Discussion

The review explores the complex interplay among epigenetic mechanisms, stress, coping strategies, and affective disorders. It synthesizes findings from 33 studies to elucidate the role of epigenetic changes induced by stressors in mood disorder occurrence, development, and progression, alongside the influence of coping mechanisms on these processes.

### 4.1. Epigenetic Mechanisms and Affective Disorders

The review highlights the significant associations between epigenetic changes in stress-related genes and the diagnosis of affective disorders. Specifically, genes, such as NR3C1, SLC6A4, BDNF, FKBP5, SKA2, and OXTR, exhibit alterations in DNA methylation patterns that are frequently linked to mood disorders. Notably, these changes often stem from early life experiences, including childhood trauma, parental abuse, or neglect. However, the relationship between specific epigenetic modifications and psychiatric symptoms is nuanced, with contradictory findings observed in some studies.

NR3C1 (human glucocorticoid receptor gene):

Studies by Bustamante et al. [21], Radtke et al. [22], Perroud et al. [23], and De Assis Pinheiro et al. [24] have reported associations between NR3C1 methylation and childhood trauma, major depressive disorder, and other psychiatric symptoms. Methylation patterns in NR3C1 have been linked to changes in gene expression and the stress response. Bustamante et al. [21] reported significantly higher methylation levels of NR3C1 in individuals with a history of childhood trauma and recurrent depressive disorder, suggesting an epigenetic link between early-life adversity and mood disorders. Radtke et al. [22] found correlations between NR3C1 methylation and specific depressive symptoms, indicating the complex relationship between epigenetic modifications and psychiatric phenotypes. Perroud et al. [23] demonstrated positive correlations between NR3C1 methylation and childhood abuse severity in patients with major depressive disorder, further supporting the role of epigenetic changes in stress-related pathways.

FKBP5 (FK506 5 binding protein gene):

This gene plays a crucial role in regulating the stress response system, and its methylation patterns have been associated with psychiatric symptoms and resilience. Weder et al. [26] identified differences in FKBP5 methylation between individuals with and without a history of abuse. Tyrka et al. [27] reported lower methylation levels of FKBP5 in individuals with a history of childhood abuse and mood disorders, indicating complex interactions between genetic and environmental factors in stress-related disorders. Flasbeck and Brüne [28] explored the association between FKBP5 methylation and anxiety, providing insights into the gene’s involvement in emotional regulation and empathy.

SLC6A4 (serotonergic transporter gene):

Alterations in SLC6A4 methylation have been linked to changes in serotonin signaling and mood regulation. Sanwald et al. [29] and Swartz et al. [30] investigated SLC6A4 methylation in the context of depression severity and socioeconomic stress, revealing potential epigenetic mechanisms underlying mood disorders. Booji et al. [31] observed correlations between SLC6A4 methylation and childhood abuse history, suggesting a link between early-life stressors and serotonin system dysregulation.

BDNF (brain-derived neurotrophic factor):

Lower methylation levels of BDNF have been associated with increased symptom severity and susceptibility to stress-induced mood disorders. Song et al. [35] reported associations among BDNF methylation, depressive symptoms, and work stress levels, indicating the gene’s involvement in stress-related psychiatric disorders.

SKA2 (kinetochore protein gene):

SKA2 methylation patterns have been linked to stress response dysregulation and mood disorder pathophysiology. Studies by Sadeh et al. [36,37] investigated SKA2 methylation in relation to PTSD and depressive symptoms, highlighting its potential as a biomarker for stress-related psychiatric disorders.

OXTR (oxytocin receptor):

The dysregulation of OXTR methylation has been associated with impaired social functioning and emotional processing. Smearman et al. [38] and Ludwig et al. [40] explored OXTR methylation in the context of childhood abuse and depression severity, providing insights into the gene’s role in social functioning and emotional regulation. Reiner et al. [41] reported lower OXTR methylation levels in depressed patients compared to healthy controls, suggesting alterations in oxytocin signaling pathways in mood disorders.

### 4.2. Coping Mechanisms and Affective Disorders

The review discusses the role of coping strategies in modulating the severity and recurrence of affective disorders. Emotion-focused coping, characterized by strategies aimed at managing emotional distress, shows mixed associations with symptom severity. While some studies suggest that emotion-focused coping exacerbates depressive or manic episodes, others report no significant impact [43,44]. Conversely, active coping strategies, such as problem-solving and seeking social support, are correlated with better outcomes, including more extended remission periods and lower recurrence rates [45,47]. However, the distinction between adaptive and maladaptive coping strategies is complex, and longitudinal studies are needed to delineate their predictive value in affective disorders.

### 4.3. Diagnosis-Specific Insights into Affective Disorders

A nuanced understanding of the interplay among epigenetic mechanisms, stressors, coping strategies, and affective disorders reveals diagnosis-specific insights that shed light on the complex nature of mood disorders. Here, we delve into the diagnosis-specific findings and implications highlighted by the reviewed studies:

Major Depressive Disorder (MDD):

Studies focusing on MDD underscore the intricate relationship among early-life adversity, epigenetic modifications, and the development of depressive symptoms [21]. NR3C1 methylation emerges as a common theme, with some studies reporting higher methylation levels associated with childhood trauma and recurrent depression, while others found lower methylation levels in individuals with mood disorders [21,22]. SLC6A4 methylation also shows associations with depressive symptom severity, suggesting the involvement of serotonin system dysregulation in MDD pathogenesis [29]. Coping mechanisms play a crucial role in modulating symptom severity and recurrence in MDD, with active coping strategies correlated with better outcomes [44].

Bipolar Disorder (BD):

Research on BD highlights the heterogeneity of coping responses and stress susceptibility across different mood states [45]. FKBP5 and OXTR methylation patterns demonstrate associations with BD susceptibility and symptom severity, providing insights into the biological underpinnings of the disorder [25,40]. Coping strategies in BD exhibit distinct patterns compared to MDD, with problem-focused coping showing correlations with better functionality and resilience [47].

Post-Traumatic Stress Disorder (PTSD):

Studies investigating PTSD reveal the enduring impact of trauma on epigenetic regulation and stress response pathways [38]. SKA2 methylation emerges as a potential biomarker for PTSD, with alterations in the gene’s methylation levels associated with symptom severity [36]. Coping mechanisms in PTSD reflect maladaptive responses to trauma exposure, highlighting the need for targeted interventions to address stress-related symptoms effectively.

Generalized Anxiety Disorder (GAD):

While fewer studies focus specifically on GAD, findings suggest associations between NR3C1 and FKBP5 methylation and anxiety symptomatology. Coping strategies in GAD emphasize the role of emotion-focused coping in exacerbating symptoms, underscoring the need for interventions targeting adaptive coping mechanisms [51].

### 4.4. Clinical Implications and Future Directions

Clinical Implications:

Early Intervention Strategies: Research has highlighted the importance of early intervention strategies in mitigating the impact of stress-induced epigenetic changes on mental health outcomes. The early identification of individuals with a history of childhood trauma or adverse experiences could facilitate targeted interventions to prevent the development of affective disorders [21,22].

Personalized Treatment Approaches: Understanding the role of epigenetic modifications in stress-related genes offers opportunities for personalized treatment approaches. Tailoring interventions based on an individual’s epigenetic profile could enhance treatment efficacy and optimize outcomes in patients with mood disorders [23].

Risk Assessment and Prevention: Epigenetic biomarkers associated with stress-related genes could serve as valuable tools for risk assessment and prevention strategies. Screening individuals for aberrant DNA methylation patterns linked to increased susceptibility to affective disorders could enable the early identification of at-risk populations and the implementation of preventive interventions [25,28].

Targeted Pharmacotherapy: Insights into epigenetic regulation offer opportunities for the development of targeted pharmacotherapeutic interventions. Drugs modulating DNA methylation or histone modifications could influence gene expression profiles implicated in mood disorders, providing novel avenues for pharmacological interventions [29,31].

Future Directions:

Longitudinal Studies: Longitudinal studies are essential to elucidate the dynamic nature of epigenetic changes in response to stressors and their impact on affective disorders. The long-term follow-up of individuals exposed to early-life adversity could shed light on the persistence and stability of epigenetic modifications over time [25,27].

Translational Research: Bridging the gap between preclinical research and clinical practice is crucial for translating findings into clinical applications. Translational research efforts aimed at validating epigenetic biomarkers in clinical settings and assessing their utility for diagnosis, prognosis, and treatment monitoring are warranted [32,37].

Interventional Studies: Interventional studies evaluating the efficacy of interventions targeting epigenetic mechanisms for the prevention and treatment of affective disorders are needed. Randomized controlled trials investigating the effects of lifestyle modifications, pharmacological agents, or psychosocial interventions on epigenetic markers and clinical outcomes could provide valuable insights into novel therapeutic approaches [33,34].

Multi-omic Approaches: The integration of epigenomic data with other omics layers, such as genomics, transcriptomics, and proteomics, could enhance our understanding of the molecular mechanisms underlying affective disorders. Multi-omic approaches could elucidate complex gene–environment interactions and identify novel therapeutic targets for intervention [40].

Understanding the intricate relationship between epigenetic mechanisms, stress, coping mechanisms, and affective disorders has important clinical implications [52,53,54]. Targeted interventions that address genetic predisposition and environmental stressors could lead to more effective treatment strategies. Longitudinal studies are needed to further elucidate the role of coping mechanisms in the onset, recurrence, and progression of affective disorders [44,45]. Additionally, future research should focus on identifying specific genomic loci involved in affective disorders and exploring the clinical implications of coping strategies in therapeutic settings [1].

The study has some limitations:

- Heterogeneity of study designs: The review encompasses studies with varying methodologies, including cross-sectional and longitudinal designs, which may limit the generalizability of the findings.
- Sample characteristics: Studies included in the review involve diverse patient populations with variations in age, gender, and clinical characteristics, potentially confounding the interpretation of results.
- Measurement of epigenetic changes: The review primarily focuses on DNA methylation as a proxy for epigenetic alterations, overlooking other mechanisms, such as histone modifications or microRNA regulation, which could also contribute to the pathophysiology of affective disorders.
- Causality and directionality: Most reviewed studies establish associations among epigenetic changes, stressors, coping mechanisms, and affective disorders, but the causality and directionality remain unclear. Longitudinal studies are necessary to elucidate temporal relationships and causal pathways.
- Publication bias: The review may be subject to publication bias, as studies reporting statistically significant findings are more likely to be published, potentially skewing the evidence synthesis.

Further research should address the limitations mentioned above by employing longitudinal designs, integrating multi-omic approaches to explore comprehensive epigenetic mechanisms, and examining diverse coping strategies across different stages of affective disorders. Additionally, translational studies are warranted to translate research findings into personalized interventions and therapeutic approaches for individuals with mood disorders [1,12].

## 5. Conclusions

The review underscores the intricate interplay among epigenetic processes, stress responses, coping mechanisms, and affective disorders, providing valuable insights into the underlying mechanisms and avenues for future investigation and clinical intervention.

Despite challenges and limitations, understanding these interactions is critical for advancing our knowledge of mood disorders and developing more targeted and personalized treatment approaches.

## Figures and Tables

**Table 1:** Stress-related genes and their association with the presence of an affective disorder.

Gene	Study	Stress	Affective Disorder	Questionnaire
NR3C1	Bustamante et al. [21]	Positive association	Negative association	CTS (child trauma screen), CTQ (childhood trauma questionnaire)
	Radtke et al. [22]	Did not report	Did not report	KERF 1
	Perroud et al. [23]	Positive association	Did not report	CTQ
	De Assis Pinheiro et al. [24]	Negative association	Positive association	BDI-II (Beck Depression Inventory II)
	Comtois-Cabana et al. [25]	Positive association	Negative association	CTQ-SF (child trauma screen—short form), BDI-II
FKPB 5	Weder et al. [26]	Positive association	Negative association	CTQ
	Tyrka et al. [27]	Negative association	Positive association	Interview
	Flasbeck & Brüne [28]	Negative association	Positive association	CTQ
SLC6A4	Sanwald et al. [29]	Positive association	Positive association	MADRS (Montgomery–ÅsbergDepression Rating Scale)
	Swartz et al. [30]	Positive association	Positive association	Interview
	Booji et al. [31]	Positive association	Did not report	CTQ
	Lei et al. [32]	Positive association	Positive association	Non-standard 11 item scale
	Kang et al. [33]	Positive association	Did not report	Non-standard childhood adversity
	Alaasari et al. [34]	Negative association	Positive association	Karasek-Job Content Questionnaire
	Comtois-Cabana et al. [25]	Positive association	Positive association	CTQ-SF, BDI-II
BDNF	Song et al. [35]	Positive association	Negative association	Non-standard self-report questionnaire
SKA2	Weder et al. [26]	Did not report	Did not report	Report of parental abuse and neglect
	Sadeh et al. [36]	Did not report	Positive association	PTSD (post-traumatic stress disorder) scale administered by clinicians
	Sadeh et al. [37]	Positive association	Did not report	PTSD scale administered by clinicians
OXTR, LINGO3, POU3F1, ITGB1.	Smearman et al. [38]	Positive association	Did not report	CTQ
	Lutz et al. [39]	Positive association	Negative association	CECA (Childhood Experience of Care and Abuse), Interview
	Ludwig et al. [40]	Did not report	Positive association	CTQ, HAM-D (Hamilton Depression Rating Scale)
	Kogan et al. [41]	Positive association	Positive association	ACE (Adverse Childhood Experiences)

**Table 2:** Coping mechanisms in patients with bipolar disorder and major depressive disorder.

Study	Number of Patients	Gender	Age (Average)	Questionnaire	Results
Kasi et al. [43]	162, MDD (major depressive disorder) and GAD (generalized anxiety disorder)	74.4% M; 25.3% F	It does not specify	COPE (Coping Orientation to Problems Experienced Inventory)	In patients diagnosed with generalized anxiety disorder or major depressive disorder, “religion-oriented” was the most common coping mechanism identified.
Horwitz et al. [44]	286, MDD	41% M; 59% F	18	COPE, C-SSRS (Columbia Suicide Severity Rating Scale)	Active coping was correlated with lower C-SSRS sores at follow-up.
Fletcher et al. [45]	379, BD (bipolar disorder) + MDD	41% M; 59% F	39	COPE, RPA (Responses to Positive Affect), CIPM (Coping Styles in Prodrome of Bipolar Mania), RSQ (Response Style Questionnaire), CERQ (Cognitive Emotion Regulation Questionnaire)	A number of differences were found between the group of patients with unipolar depression and the group with bipolar depression, the former being oriented towards active coping, focused on the problem.
Au CH et al. [46]	115, BD	37% M; 63% F	47	SCOS (Stigma Coping Orientation Scale)	It has been reported that low self-esteem is crucial for social functioning. Dysfunctional coping predominates among these patients.
Nitzburg et al. [47]	92, BD	48% M; 42% F	45	COPE	Dysfunctional coping is a predictive factor for many disabilities, while active coping is associated with resilience. Likewise, behavioral disengagement and guilt are predictors of disability.
Paans et al. [48]	90, BD	45% M; 55% F	67	UCL	The authors reported positive associations between better cognitive functioning and active coping.
Lin J et al. [7]	310, MDD with suicidal risk	It does not specify	30	SCSQ (Simplified Coping Style Questionnaire)	Patients at risk of suicide had negative coping strategies and an inadequate social support network.
Kuiper et al. [49]	89, MDD	It does not specify	20	COPE	Problem-centered coping has been shown to correlate with better functionality. Emotion-centered coping and dysfunctional coping have been associated with low resilience
Orzechowska et al. [50]	80, MDD and BD	48 women, 32 men	49	COPE	Unlike healthy people, depressed patients in stressful situations more often use strategies based on avoidance and denial and have more difficulty in finding positive aspects of stressful events.
Roohafza HR et al. [51]	4685, MDD and GAD	It does not specify	49	COPE	The results show that positive interpretation and growth, active coping, and a supportive social network are protective factors in major depressive disorder and generalized anxiety disorder.

## Data Availability

Not applicable.
